# Predominantly left-lateralized EEG–EMG associations following gamma–theta stimulation in older adults with mild cognitive impairment

**DOI:** 10.3389/fnagi.2026.1882681

**Published:** 2026-07-17

**Authors:** Runhong Yao, Kouji Yamada, Masahiro Kudo, Duojin Wang

**Affiliations:** 1 Faculty of Health Sciences, Nihon Institute of Medical Science, Irumagun, Saitama, Japan; 2 Graduate School of Health Sciences, Fujita Health University, Toyoake, Aichi, Japan; 3 Institute of Rehabilitation Engineering and Technology, University of Shanghai for Science and Technology, Shanghai, China

**Keywords:** aging neuroscience, EEG–EMG coupling, functional connectivity, gamma–theta stimulation, mild cognitive impairment, neural oscillations, neuromodulation, trunk muscles

## Abstract

**Introduction:**

Mild cognitive impairment (MCI) is frequently accompanied by impairments in postural control and trunk muscle function. Although gamma–theta rhythmic stimulation has been reported to modulate neural oscillatory activity, its influence on brain–muscle interactions in individuals with MCI remains poorly understood. This exploratory pilot study investigated whether changes in EEG connectivity following gamma–theta stimulation were associated with alterations in trunk muscle electromyographic (EMG) activity in older adults with MCI.

**Methods:**

Nine older adults with MCI underwent two separate 10-min sessions of combined 40 Hz visual flicker and theta-frequency mechanical stimulation. Resting-state EEG and trunk muscle EMG were assessed before and after stimulation. EEG connectivity changes were calculated using Spearman correlations between predefined electrode pairs. Supplementary analyses using phase-lag index (PLI) and imaginary coherence (iCoh) were performed to address potential volume conduction effects. EMG activity was recorded bilaterally from the multifidus and lumbar paraspinal muscles during trunk extension tasks. Correlation analyses examined associations between EEG connectivity changes and EMG changes in RMS amplitude, mean frequency, and spectral power measures.

**Results:**

Although no predefined EEG connectivity pair demonstrated significant group-level change following stimulation, several EEG connectivity change scores were significantly associated with concurrent EMG changes. The most robust associations involved left multifidus activity: FC5–F3 connectivity changes were associated with left multifidus RMS amplitude (*ρ* = +0.800, *p* = 0.010), and T7–T8 connectivity changes were associated with left multifidus mid-frequency spectral power (*ρ* = −0.800, *p* = 0.010). Leave-one-out sensitivity analyses indicated that both associations remained statistically significant in all nine subsets. Supplementary phase-based connectivity analyses provided limited convergent support for the primary findings. Although several contralateral EEG–EMG associations were identified, the overall pattern was characterized by a predominance of ipsilateral associations.

**Discussion:**

These findings suggest that gamma–theta stimulation may influence coordinated brain–muscle responses in older adults with MCI, with a predominance of left-hemisphere EEG–EMG associations indicating relative hemispheric predominance rather than strict hemispheric exclusivity. Associations involving broader measures of muscle activation and spectral distribution were more robust than those involving specific spectral indices. Given the small sample size, absence of sham control, non-simultaneous EEG–EMG recording, and exploratory statistical approach, these findings should be interpreted as hypothesis-generating and require confirmation in larger controlled studies.

## 1 Introduction

MCI is widely recognized as a transitional stage between normal aging and dementia and affects approximately 10–20% of adults over 65 years of age. Although MCI is primarily characterized by cognitive decline, growing evidence suggests that motor dysfunction frequently accompanies cognitive impairment in this population (Petersen et al., 2009; Albert et al., 2011; Jack et al., 2018). Individuals with MCI often exhibit impairments in gait, balance, postural stability, and trunk control, even during the early stages of disease progression (Montero-Odasso et al., 2012, 2014; Beauchet et al., 2016). These observations suggest that neural alterations associated with MCI extend beyond cognitive networks and may also affect systems involved in motor control and sensorimotor integration.

Brain–muscle interactions are thought to play a central role in the coordination of posture and movement. Functional relationships between cortical activity and peripheral muscle activation have traditionally been investigated using electroencephalography (EEG) and EMG, particularly through measures of cortico-muscular coupling and coherence (Mima and Hallett, 1999; Baker, 2007; Kristeva et al., 2007; Ushiyama et al., 2017). These studies have shown that coherent oscillatory activity between motor cortex and peripheral muscles, predominantly in the beta and gamma frequency bands, reflects functional corticospinal communication during voluntary motor tasks. Previous studies have demonstrated that alterations in brain–muscle interactions occur in aging and neurological disorders, including stroke, Parkinson’s disease, and age-related motor decline (Sailer et al., 2000; Rossiter et al., 2013). However, relatively little is known about how brain–muscle relationships respond to sensory neuromodulation in older adults with cognitive impairment. In particular, it remains unclear whether stimulation-induced changes in large-scale cortical networks are associated with corresponding changes in trunk muscle activity.

Neural oscillations provide a potential mechanism linking cognitive and motor systems. Gamma oscillations (~30–80 Hz) are associated with local neuronal synchronization and higher-order cognitive functions, whereas theta oscillations (4–8 Hz) contribute to long-range communication between distributed neural networks (Buzsáki, 2002; Fries, 2005; Buzsáki and Moser, 2013). Interactions between these rhythms occur through cross-frequency coupling (CFC), particularly theta–gamma coupling, which has been proposed as a key mechanism supporting memory encoding and information integration (Canolty and Knight, 2010; Lisman and Jensen, 2013). Altered theta–gamma coupling has been reported in both Alzheimer’s disease and MCI, suggesting that disrupted oscillatory coordination may represent an early feature of neurodegeneration (Goodman et al., 2018; Brooks et al., 2024). An additional consideration in the interpretation of oscillatory responses to neuromodulation is hemispheric asymmetry. Functional and structural differences between the two cerebral hemispheres can influence responsiveness to external stimulation (Schade et al., 2012; Mazoyer et al., 2014; Konieczny et al., 2022). In right-handed individuals, the left hemisphere is typically dominant for motor planning and execution (Springer et al., 1999; Knecht et al., 2000), and there is evidence that the two hemispheres may differ in their susceptibility to both neuromodulatory interventions and neurodegeneration (Di Lazzaro et al., 1999; Schade et al., 2012).

Recent neuromodulation studies have demonstrated that rhythmic sensory stimulation can entrain neural oscillations. Gamma-frequency visual stimulation has been shown to modulate cortical activity and reduce amyloid-related pathology in animal models (Iaccarino et al., 2016; Adaikkan et al., 2019), while theta-frequency sensory stimulation can influence sensorimotor oscillatory dynamics (Spooner et al., 2022). These findings raise the possibility that combined gamma–theta stimulation may influence both cortical network organization and motor-related neural processes. Trunk muscles, including the multifidus and lumbar paraspinal muscles, are of particular interest because of their critical role in postural control and spinal stability, functions that are frequently compromised in older adults with cognitive impairment (Beauchet et al., 2009; Chimsuwan et al., 2024).

Despite increasing interest in gamma- and theta-based neuromodulation, little is known about whether stimulation-induced changes in cortical network activity are associated with peripheral motor adaptations in individuals with MCI. Resting-state functional connectivity may provide a useful framework for characterizing stimulation-related reorganization of distributed cortical networks beyond local oscillatory power changes (Fox and Raichle, 2007; Bassett and Sporns, 2017; Yao et al., 2026).

Therefore, the present exploratory pilot study investigated whether changes in resting-state EEG functional connectivity following polyrhythmic gamma–theta stimulation were associated with changes in trunk muscle activity in older adults operationally classified as having MCI. We further explored whether these associations demonstrated patterns of hemispheric predominance and whether they remained robust across sensitivity analyses. We hypothesized that stimulation-related changes in inter-regional EEG connectivity would be associated with changes in EMG-derived measures of trunk muscle activity, particularly within frontal–motor and temporal networks involved in postural regulation.

## 2 Methods

### 2.1 Participants

Nine older adults participated in this exploratory pilot study (age: 72.4 ± 5.3 years; 5 females, 4 males). Participants were recruited from daycare facilities and were operationally classified as having MCI on the basis of Mini-Mental State Examination (MMSE) scores ranging from 22 to 26 (mean: 24.1 ± 1.4), consistent with prior observational studies in community-dwelling older adults. We note that this operational definition did not include confirmatory neuropsychological testing, such as the Montreal Cognitive Assessment or logical memory subtests, nor formal structured screening for depression or delirium. Accordingly, the cohort should be considered a clinically defined sample rather than a research-diagnostic MCI group meeting the full Petersen criteria (Petersen, 2004). Participants with known photosensitivity, seizure history, or uncorrected visual impairment that would preclude participation in the visual stimulation protocol were excluded.

All participants were community-dwelling older adults who were able to walk independently and perform standing trunk extension movements without physical assistance. Independent ambulation was defined as Functional Ambulation Category ≥ 4. Handedness was assessed using the Edinburgh Handedness Inventory (Oldfield, 1971), and all participants were classified as right-handed.

All participants provided written informed consent before participation. The study protocol was approved by the Ethics Committee of Japan University of Health Sciences (Approval No. P2103) and was conducted in accordance with the principles of the Declaration of Helsinki.

### 2.2 Intervention

Participants received two separate 10-min sessions of polyrhythmic gamma–theta cross-frequency coupling stimulation using a custom-built polyrhythmic stimulation system.

The experimental timeline consisted of: (1) baseline EEG and EMG assessments; (2) a first 10-min stimulation session; (3) post-stimulation EMG assessment immediately after the first session; (4) a 3-h interval; (5) a second identical 10-min stimulation session; and (6) post-stimulation EEG assessment immediately after the second session.

EMG was assessed immediately after the first stimulation session to capture acute changes in trunk muscle activity. EEG was assessed after the second stimulation session to minimize contamination of EEG recordings by movement-related and electromyographic artefacts associated with trunk extension testing. Because the two stimulation sessions were identical in parameters and duration, observed EEG and EMG changes were interpreted as reflecting responses to equivalent stimulation exposures measured at different time points.

The 3-h inter-session interval was informed by evidence that acute aftereffects of repetitive sensory stimulation typically resolve within 60–90 min (Ridding and Rothwell, 2007; Nitsche et al., 2008; Suppa et al., 2016); its adequacy for gamma–theta stimulation in MCI remains unknown and is discussed as a limitation.

The stimulation paradigm combined two synchronized modalities. Visual gamma stimulation was delivered using a Gamma Light Therapy device (GLS-40, Gamma Light Therapy LLC, USA), producing a 40-Hz flickering light stimulus at an illuminance of approximately 300 l× at a viewing distance of 30 cm. Mechanical theta stimulation was delivered using a vibration platform (WBN5020K, Alinco, China) equipped with an oscillatory motor (FAV5019N), producing vertical oscillatory movement with an amplitude of approximately 10 mm at a frequency centred around 5 Hz.

The two components were temporally synchronized so that approximately eight 40-Hz gamma cycles occurred within each 5-Hz theta cycle, mimicking endogenous theta–gamma cross-frequency interactions. Participants remained seated and were instructed to stay relaxed without performing voluntary movements.

### 2.3 EEG recording and analysis

Neural oscillatory activity was recorded using a wearable 14-channel EEG system (EMOTIV EPOC X, Emotiv Inc., San Francisco, CA, USA) with electrodes positioned according to the international 10–20 system at AF3, AF4, F3, F4, F7, F8, FC5, FC6, T7, T8, P7, P8, O1, and O2. The system uses the manufacturer-defined CMS/DRL reference configuration. Signals were sampled at 256 Hz.

EEG recordings were obtained during eyes-open resting-state conditions immediately before and after stimulation, with eeach recording lasting 180 s. Raw EEG signals were band-pass filtered between 0.5 and 45 Hz and visually inspected for artefacts. Segments containing excessive noise or peak-to-peak amplitudes exceeding ±150 μV were excluded from further analysis.

Inter-regional EEG signal coupling was estimated using Spearman rank correlations between electrode time series, computed separately for pre- and post-intervention recordings. This approach was adopted as a simple, non-parametric index of signal co-variation suitable for analyses in a small pilot sample (Gibbons and Chakraborti, 2010; Garrocho-Rangel et al., 2024). We acknowledge that correlation-based connectivity measures do not explicitly account for volume conduction and may therefore overestimate coupling between spatially proximate electrodes. Therefore, the connectivity results should be interpreted as indices of functional signal coupling rather than direct measures of neural communication.

To address this limitation, supplementary analyses using phase-lag index (PLI) and imaginary coherence (iCoh), both of which are less sensitive to zero-lag volume conduction effects, were performed across theta (4–8 Hz) and gamma (27–45 Hz) frequency bands. These analyses are presented in the Supplementary Material.

Based on previous findings suggesting the involvement of frontal, temporal, and sensorimotor networks in gamma–theta stimulation-related modulation, six electrode pairs were selected *a priori* for analysis: F4–T8, FC6–F4, AF3–F3, FC5–F3, O1–O2, and T7–T8 (Yao et al., 2026).

Connectivity change scores were calculated as:

*Δ*r = r_post − r_pre

for each participant and electrode pair. The resulting Δr values were treated as indices of directional connectivity change rather than absolute measures of connectivity strength.

Single-channel analyses were also conducted to determine whether observed EEG–EMG associations were specific to inter-regional connectivity measures. For each electrode, the mean amplitude of the filtered EEG signal was calculated separately for pre- and post-intervention recordings, and change scores (Δ = post − pre) were subsequently correlated with EMG change variables using Spearman rank correlation analysis.

### 2.4 EMG recording and analysis

Surface EMG signals were recorded bilaterally from four trunk muscles: the left and right multifidus and the left and right lumbar paraspinal muscles. Recordings were obtained using a wireless surface EMG system (PW-WS1233, Precious Work Co., Ltd., Osaka, Japan) with disposable Ag/AgCl electrodes (H124SG, Precious Work Co., Ltd., Osaka, Japan). Electrode placement followed SENIAM recommendations, with an inter-electrode distance of approximately 20 mm in a bipolar configuration (Hermens et al., 2000). Signals were sampled at 200 Hz.

Participants performed a standardised trunk extension task in the standing position. They were instructed to stand upright and perform a gentle, submaximal trunk extension movement while maintaining the extended position for approximately 5 s. The task was repeated three times with 30-s rest intervals between trials. Standardised verbal instructions were provided by the same investigator across all sessions to ensure consistency. EMG feature values were averaged across the three valid trials for each muscle.

EMG signals were band-pass filtered using a fourth-order Butterworth filter (20–100 Hz) before feature extraction. For RMS analysis, signals were full-wave rectified and smoothed using a 100-ms moving window. Frequency-domain measures were computed from the filtered, non-rectified EMG signals using Welch’s power spectral density estimation with a Hamming window, 50% overlap, and a segment length of 512 samples.

Root mean square (RMS) amplitude was calculated as an index of overall muscle activation level. Mean frequency (MF) was calculated from the power spectral density and used as an indicator of the spectral distribution of motor unit activity. Spectral power was quantified within two frequency bands: a mid-frequency band (Mid%, 20–60 Hz) and a high-frequency band (High%, 60–100 Hz). Because signals were band-pass filtered between 20 and 100 Hz, frequencies below 20 Hz were not included in the spectral analyses. The upper boundary of 100 Hz corresponds to the Nyquist frequency at the 200-Hz sampling rate.

For each EMG variable, change scores were calculated as:

*Δ* = post − pre.

Correlation analyses were subsequently performed using RMS, MF, Mid%, and High% variables to characterise complementary aspects of trunk muscle activation and spectral distribution.

### 2.5 Statistical analysis

All statistical analyses were performed using Python (version 3.13) and SPSS Statistics (version 29; IBM Corp., Armonk, NY, USA). Given the small sample size (*N* = 9) and the non-normal distribution of several variables, non-parametric methods were used throughout.

To characterize stimulation-related changes, pre–post differences were calculated for all EEG and EMG variables as:

Δ = post − pre.

For EEG connectivity analyses, connectivity change scores (Δr = r_post − r_pre) were calculated, where *r* represents the Spearman correlation coefficient calculated between predefined electrode pairs.

The primary objective of the study was to investigate whether stimulation-related changes in EEG measures were associated with changes in EMG activity. Correlation analyses were first conducted for the pre-specified EEG–EMG hypotheses. Additional analyses were subsequently performed using the full EEG–EMG correlation matrix, contralateral EEG–EMG pairings, single-channel EEG variables, and supplementary phase-based connectivity metrics (phase-lag index [PLI] and imaginary coherence [iCoh]). Spearman rank correlation coefficients (*ρ*) and associated *p*-values were calculated for EEG–EMG change-score associations.

As a sensitivity analysis, Fisher *z*-transformed connectivity change scores (Δz = z_post − z_pre) were also calculated; results are reported in Supplementary Table S5.

To evaluate the robustness of significant EEG–EMG associations, leave-one-out sensitivity analyses were performed. For each correlation of interest, analyses were repeated after sequential exclusion of a single participant. Changes in correlation magnitude and statistical significance were examined across all subsets to assess how individual observations influenced the reported findings. Associations were considered robust when statistical significance was retained in all leave-one-out iterations.

Correlation strength was interpreted according to commonly used guidelines: |*ρ*| < 0.30, weak; 0.30–0.49, moderate; 0.50–0.69, strong; and ≥ 0.70, very strong. Statistical significance was defined as *p* < 0.05 (two-tailed).

## 3 Results

### 3.1 Stimulation-related changes in EMG variables

**Figure 1:**
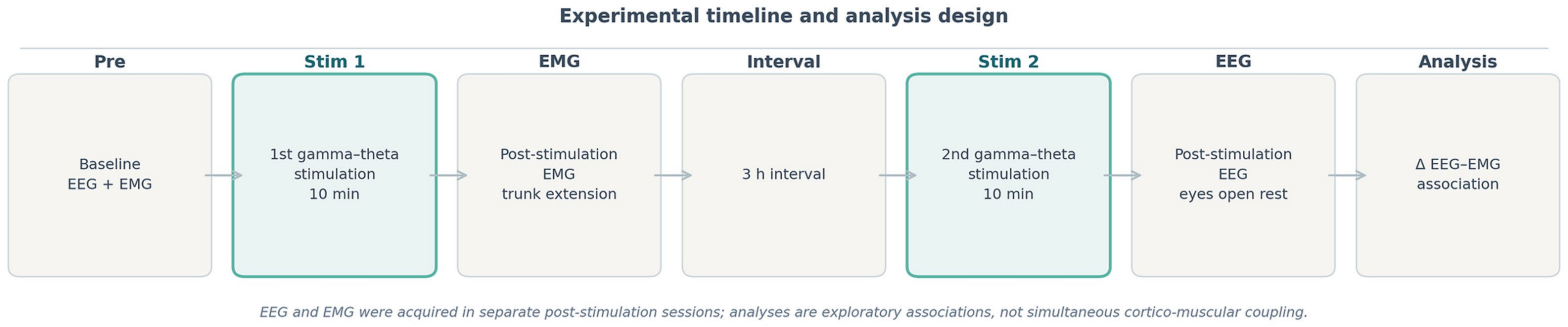
Experimental timeline and analysis design. Schematic overview of the study protocol. Participants completed baseline EEG and EMG assessments, followed by a first 10-min gamma–theta stimulation session, after which post-stimulation EMG was recorded. Following a 3-h interval, a second identical stimulation session was administered, and post-stimulation resting-state EEG was collected. Stimulation-related changes were quantified as *Δ* = post − pre for all variables. EEG and EMG were acquired in separate sessions; analyses reflect associations between stimulation-induced change scores rather than simultaneous cortico-muscular coupling.

**Figure 2:**
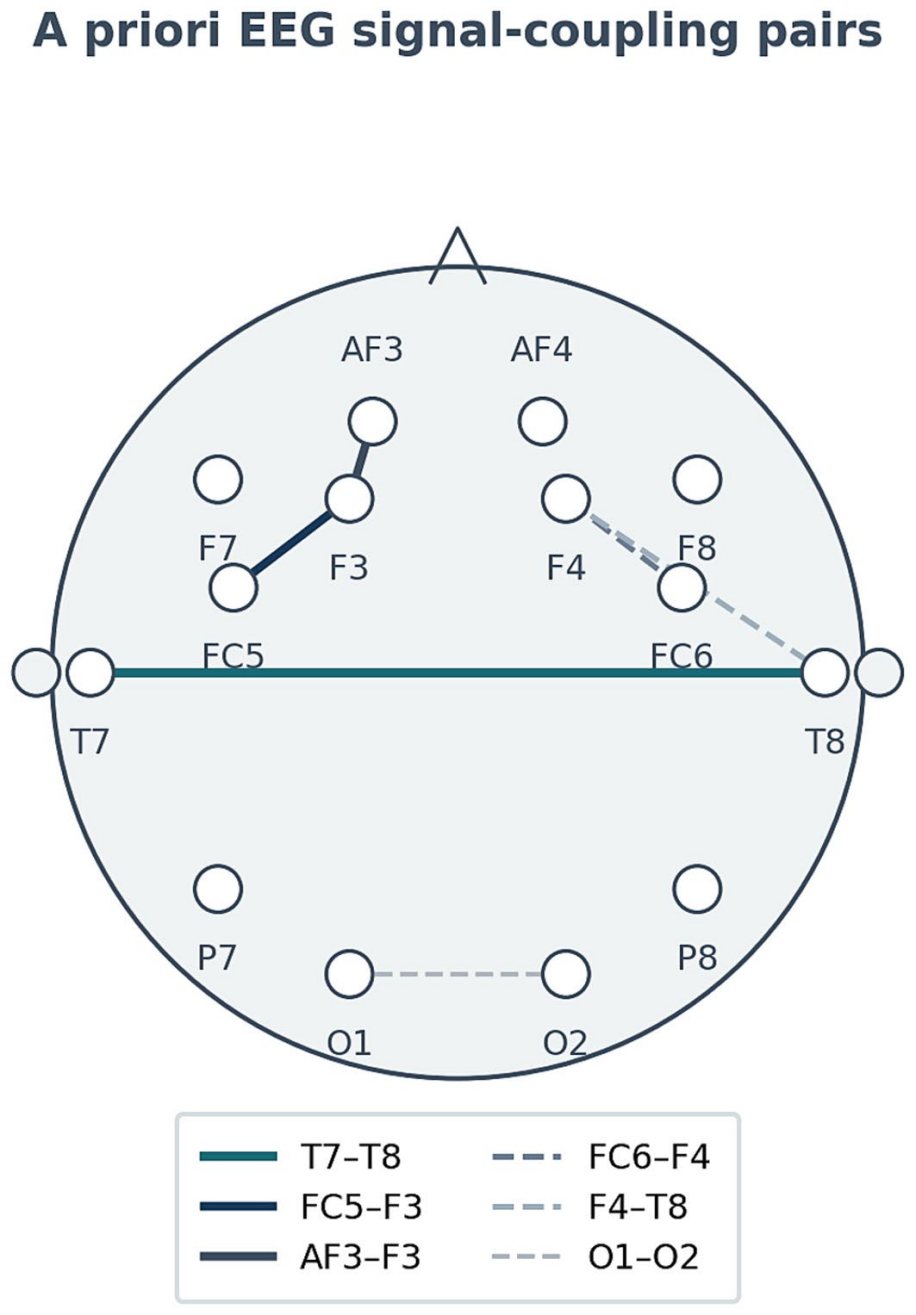
*A priori* EEG signal-coupling pairs. Scalp topographic diagram illustrating the six electrode pairs selected *a priori* for inter-regional signal coupling analysis, based on prior findings implicating frontal, temporal, and sensorimotor networks in gamma–theta stimulation responses. Electrode positions follow the international 10–20 system. T7–T8 and FC5–F3 yielded the most robust EEG–EMG associations in subsequent analyses.

Changes in trunk muscle activity following gamma–theta stimulation are summarized (Table 1). Significant post–pre reductions were observed only in the right multifidus muscle. Mean frequency decreased by 7.042 ± 5.192 Hz (Wilcoxon signed-rank test: *W* = 0, *p* = 0.004, rank-biserial correlation r_rb = 1.000), while High% spectral power decreased by 8.309 ± 9.322% (*W* = 0, *p* = 0.004, r_rb = 1.000). The effect size indicated a consistent directional response across all participants (Figures 1, 2).

**Table 1:** Changes in EMG variables following gamma–theta stimulation (Δ = post − pre, *N* = 9).

Muscle	RMS Δ (mean ± SD)	MFΔ (Hz) (mean ± SD)	Mid% Δ (%) (mean ± SD)	High% Δ (%) (mean ± SD)
R. Lumbar Paraspinals	−0.032 ± 0.186	+1.993 ± 13.494	+6.744 ± 26.568	−0.451 ± 9.677
R. Multifidus	−0.021 ± 0.214	−7.042 ± 5.192**	**−3.564 ± 13.293**	−8.309 ± 9.322**
L. Lumbar Paraspinals	−0.019 ± 0.133	+1.351 ± 5.842	+2.850 ± 13.007	+0.048 ± 6.069
L. Multifidus	+0.026 ± 0.144	−3.757 ± 12.531	+0.736 ± 15.505	−6.526 ± 11.774

RMS, root mean square amplitude; MF, mean frequency (Hz). Mid% = proportion of EMG spectral power in the 20–60 Hz band. High% = proportion of EMG spectral power in the 60–100 Hz band. Low-frequency spectral power (<20 Hz) was not analysed because EMG signals were band-pass filtered between 20 and 100 Hz. **p < 0.01 (Wilcoxon signed-rank test); right multifidus MF and High% showed significant post-stimulation decreases (both *p* = 0.004, *W* = 0, r_rb = 1.000). All other variables showed no significant pre–post differences. Large SD values reflect substantial inter-individual variability consistent with the small sample size (*N* = 9). Bold values indicate statistically significant pre–post differences (Wilcoxon signed-rank test, *p* < 0.01).

No significant changes were observed for RMS amplitude or Mid% spectral power in any muscle (all *p* > 0.05). Likewise, no significant changes were detected in the bilateral lumbar paraspinal muscles across any EMG metric.

### 3.2 Changes in EEG inter-electrode signal coupling

**Figure 3:**
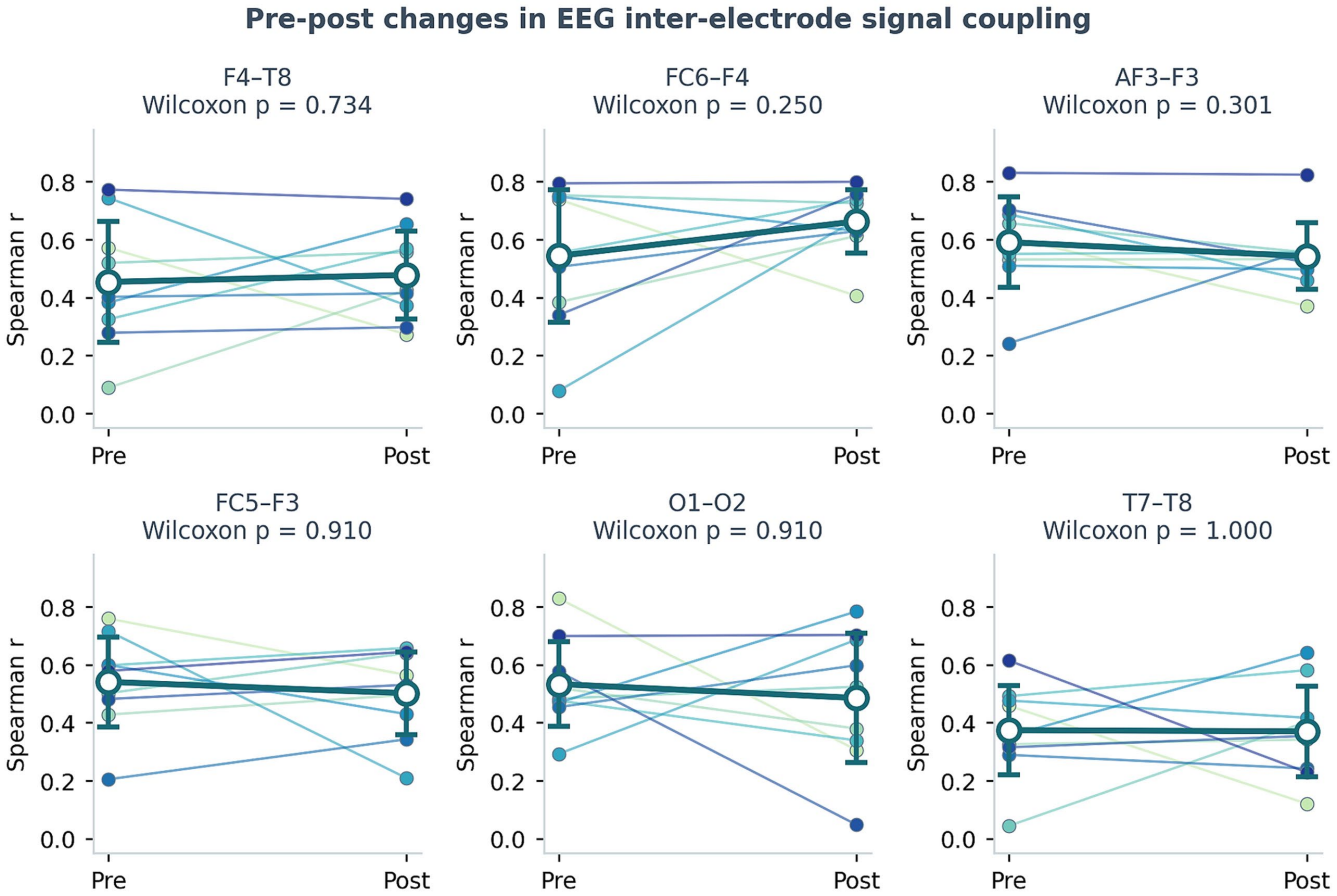
Pre- and post-intervention EEG inter-electrode signal coupling for the six predefined electrode pairs. Individual participant trajectories (thin coloured lines, *N* = 9) and group mean ± SD (filled circles with error bars, dark teal) are shown for each electrode pair. Pre-intervention (Pre) and post-intervention (Post) Spearman correlation coefficients are plotted on the y-axis. Wilcoxon signed-rank test *p*-values are indicated for each pair. No predefined connectivity pair demonstrated a statistically significant group-level change following stimulation (all *p* > 0.25). Substantial inter-individual variability was observed across all pairs. Participant colours represent individual participants (YlGnBu colour scale, consistent across all figures).

Pre- and post-intervention EEG connectivity values for the six predefined electrode pairs are presented (Table 2 and Figure 3; Supplementary Table S3).

**Table 2:** EEG inter-electrode signal coupling before and after stimulation (*N* = 9).

Electrode pair	Pre (mean ± SD)	Post (mean ± SD)	Δr (mean ± SD)	Min Δr	Max Δr
F4–T8	0.465 ± 0.222	0.477 ± 0.151	+0.022 ± 0.244	−0.370	+0.337
FC6–F4	0.550 ± 0.239	0.662 ± 0.114	+0.111 ± 0.276	−0.332	+0.591
AF3–F3	0.590 ± 0.155	0.541 ± 0.122	−0.054 ± 0.157	−0.237	+0.323
FC5–F3	0.540 ± 0.155	0.501 ± 0.143	−0.039 ± 0.201	−0.507	+0.138
O1–O2	0.533 ± 0.146	0.485 ± 0.223	−0.048 ± 0.307	−0.528	+0.395
T7–T8	0.374 ± 0.153	0.370 ± 0.157	−0.004 ± 0.233	−0.386	+0.359

Values are Spearman rank correlation coefficients computed from raw EEG voltage time series (46,080 data points per condition). Δr = r_post − r_pre. No Wilcoxon tests reached statistical significance (all *p* > 0.25). SD, standard deviation.

No predefined EEG connectivity pair demonstrated a statistically significant group-level change following stimulation (all Wilcoxon *p* > 0.25). Considerable inter-individual variability was observed, with some participants showing increases and others decreases in connectivity strength (Figure 3).

The largest mean increase was observed for FC6–F4 (Δr = +0.123 ± 0.224), whereas the largest mean decrease was observed for AF3–F3 (Δr = −0.050 ± 0.178). However, neither effect reached statistical significance (Table 3).

**Table 3:** Wilcoxon signed-rank tests: pre- vs. post-intervention comparisons (*N* = 9).

Variable	Pre (mean ± SD)	Post (mean ± SD)	Δ (mean ± SD)	*W*	*p*
EEG: F4–T8 Δr	0.465 ± 0.222	0.477 ± 0.151	+0.022 ± 0.244	20	0.820
EEG: FC6–F4 Δr	0.550 ± 0.239	0.662 ± 0.114	+0.111 ± 0.276	14	0.359
EEG: AF3–F3 Δr	0.590 ± 0.155	0.541 ± 0.122	−0.054 ± 0.157	12	0.250
EEG: FC5–F3 Δr	0.540 ± 0.155	0.501 ± 0.143	−0.039 ± 0.201	21	0.910
EEG: O1–O2 Δr	0.533 ± 0.146	0.485 ± 0.223	−0.048 ± 0.307	21	0.910
EEG: T7–T8 Δr	0.374 ± 0.153	0.370 ± 0.157	−0.004 ± 0.233	22	1.000
EMG: R. Mult. MF (Hz)	41.49 ± 14.90	34.45 ± 16.49	−7.04 ± 5.19	0	0.004**
EMG: R. Mult. High% (%)	26.29 ± 17.15	17.64 ± 16.93	−8.65 ± 9.32	0	0.004**
EMG: R. Mult. RMS	0.082 ± 0.176	0.061 ± 0.094	−0.021 ± 0.214	15	0.426
EMG: R. LP High% (%)	12.76 ± 16.96	12.31 ± 12.95	−0.451 ± 9.68	21	0.910
EMG: L. Mult. MF (Hz)	37.21 ± 16.70	33.45 ± 14.60	−3.76 ± 12.53	15	0.426
EMG: L. Mult. High% (%)	22.21 ± 15.60	15.68 ± 12.95	−6.53 ± 11.77	11	0.203
EMG: L. Mult. Mid% (%)	39.89 ± 17.85	40.63 ± 16.81	+0.74 ± 15.51	22	1.000
EMG: L. Mult. RMS	0.101 ± 0.068	0.127 ± 0.120	+0.026 ± 0.144	20	0.820
EMG: L. LP High% (%)	15.34 ± 16.13	15.38 ± 15.11	+0.048 ± 6.07	22	1.000

*W* = Wilcoxon test statistic. **p < 0.01. No EEG connectivity pairs showed significant pre–post differences (all *p* > 0.25). Right multifidus mean frequency and high-frequency power showed significant post-intervention decreases.

Given the absence of consistent group-level connectivity changes, subsequent analyses focused on whether individual differences in EEG connectivity change were associated with concurrent changes in EMG variables.

### 3.3 EEG–EMG associations following gamma–theta stimulation

Correlation analyses were first conducted for the 11 pre-specified EEG–EMG hypotheses (Table 4A).

**Table 4:** Spearman rank correlations: EEG connectivity changes vs. EMG changes (*N* = 9).

Hypothesis	EEG variable	EMG variable	*ρ*	*p*
[A] Pre-specified hypotheses (11 pairs)				
Temporal connectivity ↔ L. Mult. MF	T7–T8 Δr	L. Mult. MF Δ	**−0.683**	0.042*
Motor-frontal connectivity ↔ L. Mult. High%	FC5–F3 Δr	L. Mult. High% Δ	**−0.667**	0.050*
Motor-frontal connectivity ↔ L. Mult. MF	FC5–F3 Δr	L. Mult. MF Δ	**−0.683**	0.042*
R. Motor-frontal connectivity ↔ R. Mult. High%	FC6–F4 Δr	R. Mult. High% Δ	+0.233	0.546
F4–T8 connectivity ↔ R. Mult. High%	F4–T8 Δr	R. Mult. High% Δ	−0.250	0.516
F4–T8 connectivity ↔ R. LP High%	F4–T8 Δr	R. LP. High% Δ	0.000	>0.999
AF3–F3 connectivity ↔ L. Mult. High%	AF3–F3 Δr	L. Mult. High% Δ	−0.333	0.381
AF3–F3 connectivity ↔ L. LP High%	AF3–F3 Δr	L. LP. High% Δ	+0.033	0.932
Occipital connectivity ↔ L. Mult. RMS	O1–O2 Δr	L. Mult. RMS Δ	+0.183	0.637
Occipital connectivity ↔ R. Mult. RMS	O1–O2 Δr	R. Mult. RMS Δ	−0.033	0.932
Temporal connectivity ↔ R. Mult. MF	T7–T8 Δr	R. Mult. MF Δ	+0.050	0.898
[B] Exploratory significant associations (full 6 × 16 matrix)				
Temporal connectivity ↔ L. Mult. Mid%	T7–T8 Δr	L. Mult. Mid% Δ	**−0.800**	0.010**
Motor-frontal connectivity ↔ L. Mult. RMS	FC5–F3 Δr	L. Mult. RMS Δ	**+0.800**	0.010**
R. Motor-frontal ↔ L. LP MF [contralateral]	FC6–F4 Δr	L. LP. MF Δ	**+0.800**	0.010**
R. Motor-frontal ↔ L. LP High% [contralateral]	FC6–F4 Δr	L. LP. High% Δ	**+0.700**	0.036*
[C] Additional contralateral associations (trend-level)				
Motor-frontal ↔ R. Mult. High% [L → R contra]	FC5–F3 Δr	R. Mult. High% Δ	−0.617	0.077**†**
R. Motor-frontal ↔ L. Mult. High% [R → L contra]	FC6–F4 Δr	L. Mult. High% Δ	−0.050	0.898
Temporal ↔ R. Mult. MF [contra]	T7–T8 Δr	R. Mult. MF Δ	+0.050	0.898
Motor-frontal ↔ R. Mult. MF [L → R contra]	FC5–F3 Δr	R. Mult. MF Δ	−0.500	0.170

Section A presents 11 pre-specified hypotheses based on the study objectives; the associations of T7–T8 and FC5–F3 with left multifidus MF yield identical *ρ* values (−0.683) because the two EEG rank orderings produce the same sum of squared rank differences against the EMG variable (verified from raw data). Section B presents additional significant associations identified in the full correlation matrix (6 EEG pairs × 16 EMG variables). Section C summarizes contralateral EEG–EMG associations; all remaining contralateral combinations were non-significant (all *p* > 0.15). *ρ* = Spearman rank correlation. **p < 0.01; *p < 0.05; ^†^*p* < 0.10 (trend). *p*-values are unadjusted (exploratory pilot study; see Methods 2.5). The pattern of findings is more appropriately characterised as predominantly left-lateralized associations rather than strict hemispheric specificity, given the significant contralateral findings in Section B (FC6–F4 × L. LP High%) and the additional contralateral trends in Section C. Bold values indicate statistically significant correlations (*p* < 0.05). The dagger symbol (†) indicates trend-level correlations (*p* < 0.10).

Significant negative associations were observed between temporal connectivity changes (T7–T8 Δr) and left multifidus mean frequency changes (*ρ* = −0.683, *p* = 0.042), as well as between motor–frontal connectivity changes (FC5–F3 Δr) and left multifidus mean frequency changes (*ρ* = −0.683, *p* = 0.042).

**Figure 4:**
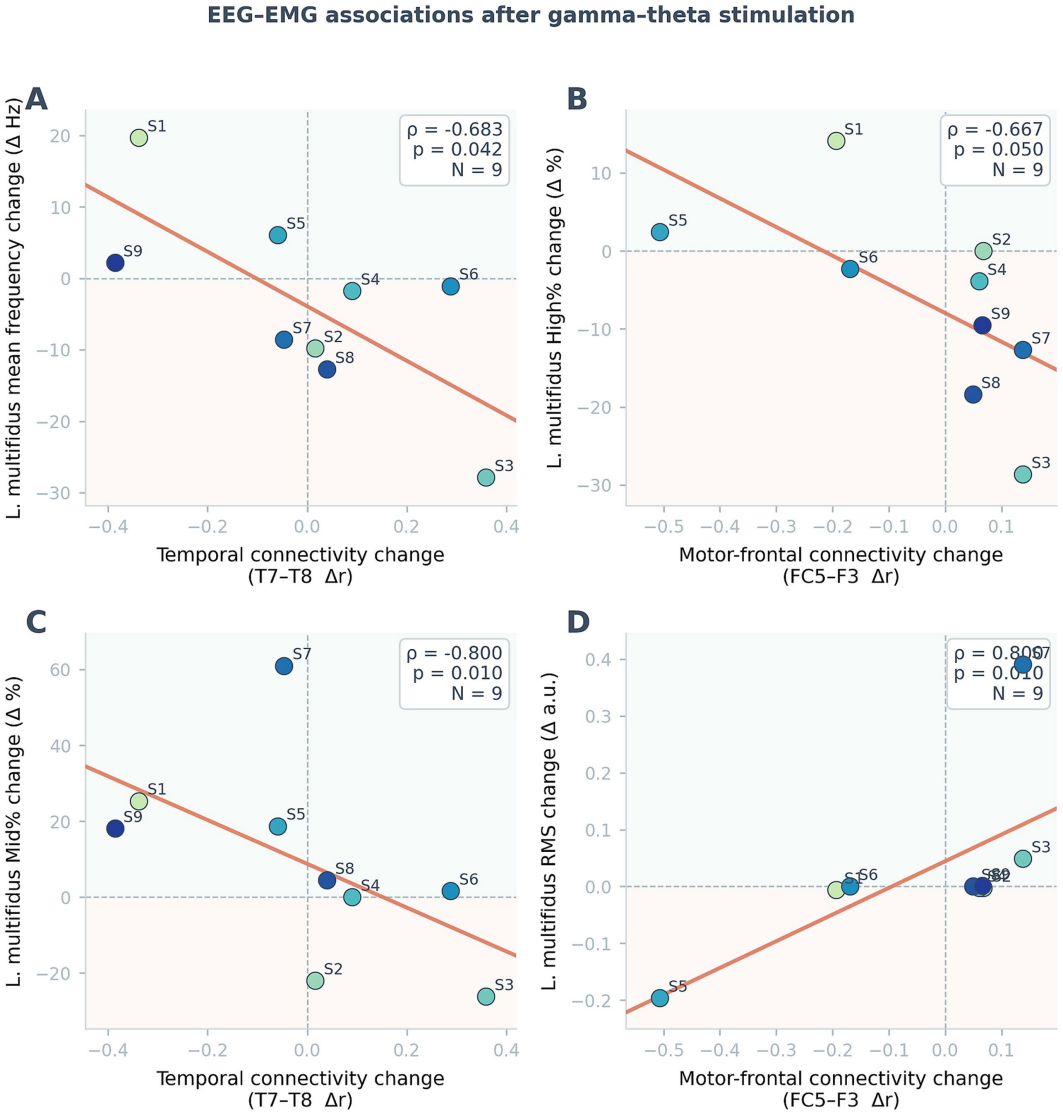
EEG–EMG associations after gamma–theta stimulation. Scatter plots showing Spearman rank correlations between EEG connectivity change scores (Δr = r_post − r_pre) and EMG change scores (Δ = post − pre) for the four principal EEG–EMG associations identified in the study. **(A)** Temporal connectivity change (T7–T8 Δr) vs. left multifidus mean frequency change (Δ Hz). **(B)** Motor-frontal connectivity change (FC5–F3 Δr) vs. left multifidus high-frequency power change (ΔHigh%). **(C)** Temporal connectivity change (T7–T8 Δr) vs. left multifidus mid-frequency power change (ΔMid%). **(D)** Motor-frontal connectivity change (FC5–F3 Δr) vs. left multifidus RMS amplitude change (ΔRMS). Each data point represents one participant (*N* = 9; YlGnBu colour scale). Dashed regression lines indicate ordinary least squares fits (shown for visual reference only; statistical analysis used Spearman rank correlation). Spearman *ρ*, *p*-values, and sample size are provided in the inset box for each panel. ** *p* < 0.01. Teal and coral shading indicate positive and negative quadrants, respectively.

A further negative association was observed between FC5–F3 connectivity changes and left multifidus High% changes (*ρ* = −0.667, *p* = 0.050) (Figures 4A,B).

Analyses of the full EEG–EMG matrix revealed several additional significant associations (Table 4B). Temporal connectivity changes (T7–T8 Δr) were strongly associated with left multifidus Mid% changes (*ρ* = −0.800, *p* = 0.010) (Figure 4C), whereas motor–frontal connectivity changes (FC5–F3 Δr) were positively associated with left multifidus RMS changes (*ρ* = +0.800, *p* = 0.010) (Figure 4D).

Additional associations were identified between FC6–F4 connectivity changes and left lumbar paraspinal MF (*ρ* = +0.800, *p* = 0.010), as well as left lumbar paraspinal High% changes (*ρ* = +0.700, *p* = 0.036).

### 3.4 Ipsilateral and contralateral EEG–EMG associations

**Figure 5:**
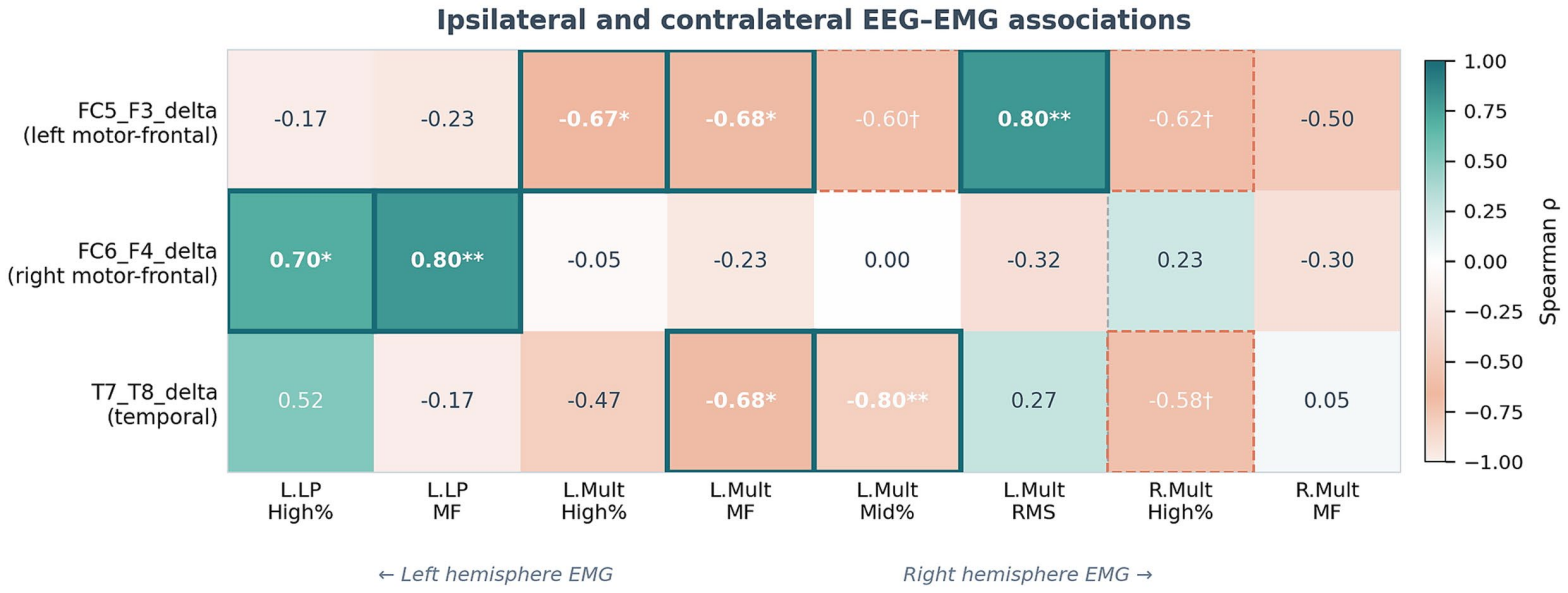
Ipsilateral and contralateral EEG–EMG associations. Heatmap showing Spearman *ρ* values for selected EEG–EMG pairings across three EEG connectivity pairs (rows) and eight EMG variables (columns). Rows represent FC5–F3 (left motor-frontal), FC6–F4 (right motor-frontal), and T7–T8 (temporal) connectivity change scores. Columns represent left lumbar paraspinal (L. LP) and left and right multifidus (L. Mult, R. Mult) EMG change variables. The first six columns represent left-hemisphere EMG variables; the final two represent right-hemisphere variables. Cell values indicate Spearman *ρ*; teal borders indicate *p* < 0.05 and dashed orange borders indicate *p* < 0.10. Cell shading reflects correlation magnitude (teal = positive, coral = negative) according to the colour scale shown. *p < 0.05; **p < 0.01. Complete results from all EEG–EMG combinations are provided in Table 4.

To further evaluate hemispheric specificity, significant EEG–EMG associations were compared across ipsilateral and contralateral pairings (Figure 5 and Table 4C).

Most significant associations involved left-hemisphere connectivity measures and left-sided trunk muscle variables. However, several contralateral relationships were also identified, including associations between FC6–F4 connectivity changes and left lumbar paraspinal variables.

Stimulation-related EEG–EMG relationships were not exclusively hemisphere-specific. Rather, the observed pattern was characterized by relative hemispheric predominance, with the most robust associations concentrated within left-sided networks, accompanied by fewer contralateral associations (Table 4C; Supplementary Table S1; Supplementary Figure S1).

Fisher *z* sensitivity analyses indicated that 7 of the 12 originally significant EEG–EMG associations remained nominally significant (Supplementary Table S5), and supplementary PLI/iCoh analyses identified significant associations involving three of the five principal electrode pairs (T7–T8, FC5–F3, and FC6–F4; Supplementary Table S4). Together, these sensitivity analyses indicate that the principal findings were not attributable to the choice of connectivity metric.

### 3.5 Leave-one-out sensitivity analysis

**Figure 6:**
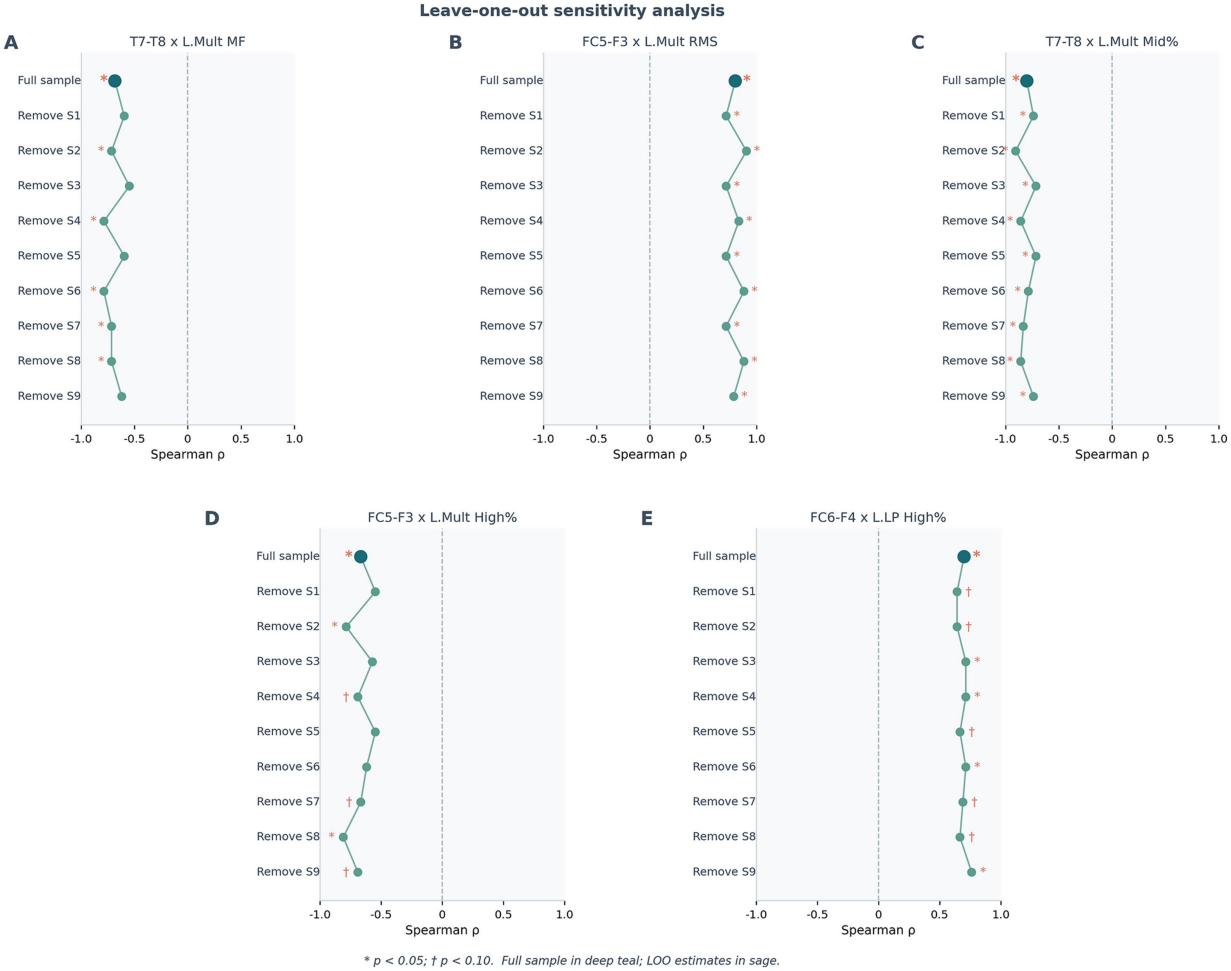
Leave-one-out sensitivity analysis for five principal EEG–EMG associations. Dot-and-line plots showing Spearman ρ for each leave-one-out (LOO) iteration and the full-sample estimate. Panels: **(A)** T7–T8 × left multifidus MF; **(B)** FC5–F3 × left multifidus RMS; **(C)** T7–T8 × left multifidus Mid%; **(D)** FC5–F3 × left multifidus High%; **(E)** FC6–F4 × left lumbar paraspinal High%. Large circles = full-sample *ρ*; small circles = LOO estimates. *p < 0.05; ^†^*p* < 0.10. Associations in **(B,C)** remained significant in all 9/9 LOO subsets; panel **(A)** in 5/9, panel **(E)** in 4/9, and panel **(D)** in 2/9 subsets, respectively (see Table 5).

To assess the robustness of the observed EEG–EMG associations, leave-one-out (LOO) sensitivity analyses were conducted for the five principal correlations (Table 5 and Figure 6).

**Table 5:** Leave-one-out sensitivity analysis for key EEG–EMG correlations.

Correlation	Full *ρ*	Full *p*	LOO max *p*	LOO *n* sig/9	Robust?
T7–T8 × L. Mult. Mid%	−0.800	0.010	0.047	9/9	Yes ✓
FC5–F3 × L. Mult. RMS	+0.800	0.010	0.047	9/9	Yes ✓
FC6–F4 × L. LP. High%	+0.700	0.036	0.086	4/9	Moderate
T7–T8 × L. Mult. MF	−0.683	0.042	0.160	5/9	Limited
FC5–F3 × L. Mult. High%	−0.667	0.050	0.160	2/9	Borderline ⚠

LOO max *p* = maximum *p*-value across 9 leave-one-out iterations. LOO *n* sig = iterations yielding *p* < 0.05. Robust = 9/9; Moderate = 4–6/9; Limited = 4–5/9; Weak ⚠ = ≤ 3/9. **p < 0.01; *p < 0.05 for full-sample correlation.

The associations between T7–T8 connectivity changes and left multifidus Mid% (*ρ* = −0.800, *p* = 0.010), and between FC5–F3 connectivity changes and left multifidus RMS (*ρ* = +0.800, *p* = 0.010), remained statistically significant in all nine leave-one-out subsets (9/9), with a maximum *p*-value of 0.047 in both cases, indicating high robustness.

The association between FC6–F4 connectivity changes and left lumbar paraspinal High% (*ρ* = +0.700, *p* = 0.036) remained significant in four of nine subsets (4/9), with a maximum *p*-value of 0.086, indicating moderate robustness.

The association between T7–T8 connectivity changes and left multifidus MF (*ρ* = −0.683, *p* = 0.042) remained significant in only five of nine subsets (5/9), with *p*-values increasing to as high as 0.160 following exclusion of certain participants.

The weakest finding was the association between FC5–F3 connectivity changes and left multifidus High% (*ρ* = −0.667, *p* = 0.050), which remained significant in only two of nine subsets (2/9), with a maximum *p*-value of 0.160.

Overall, associations involving RMS amplitude and Mid% spectral power demonstrated high robustness, while the contralateral association (FC6–F4 × L. LP High%) showed moderate stability. Associations involving MF and High% variables were more sensitive to individual observations and should be interpreted cautiously.

### 3.6 Single-channel EEG analyses

To determine whether the observed EEG–EMG relationships were specific to inter-electrode connectivity measures, correlations were performed between single-channel EEG amplitude changes and EMG change variables.

Several nominally significant associations were identified. However, these findings were distributed across isolated electrode sites and did not exhibit the coherent hemispheric organization observed in the connectivity-based analyses.

Overall, the single-channel results provided less anatomically consistent evidence for EEG–EMG coupling than the inter-regional connectivity measures (Supplementary Table S2; Supplementary Figure S2).

### 3.7 Supplementary phase-based connectivity analyses

Supplementary analyses using phase-lag index (PLI) and imaginary coherence (iCoh) were performed to evaluate the potential influence of volume conduction (Supplementary Table S4; Supplementary Figure S3).

Three of the five principal electrode pairs (T7–T8, FC5–F3, and FC6–F4) also demonstrated significant associations in the PLI/iCoh analyses, and the direction of association was consistent in all cases, suggesting that the primary findings are unlikely to be explained solely by volume conduction artifacts.

## 4 Discussion

### 4.1 Principal findings

This exploratory pilot study investigated whether changes in resting-state EEG connectivity following polyrhythmic gamma–theta stimulation were associated with changes in trunk muscle activity in older adults operationally classified as having MCI (Albert et al., 2011; Jack et al., 2018).

Three principal findings emerged.

First, although no predefined EEG connectivity pair demonstrated a significant group-level change following stimulation, several EEG connectivity change scores were significantly associated with concurrent EMG changes. This suggests that stimulation-related neural responses may have been expressed primarily through inter-individual variability rather than through uniform group-level effects (Button et al., 2013).

Second, the most robust EEG–EMG associations involved left multifidus RMS amplitude and mid-frequency spectral power, both of which remained statistically significant across all nine leave-one-out subsets. Connectivity changes in the T7–T8 and FC5–F3 electrode pairs were most consistently associated with these muscle activity variables. Both associations remained significant in all nine leave-one-out subsets (9/9).

Third, although several contralateral EEG–EMG relationships were identified, the overall pattern of findings was characterized by a predominance of ipsilateral associations. This pattern suggests relative hemispheric specialization rather than strict hemispheric exclusivity in the neural regulation of trunk muscle activity following stimulation (Knecht et al., 2000; Strutton et al., 2004). Hemispheric predominance was inferred from the concentration of the most robust and reproducible associations within left-sided networks—specifically the 9/9 leave-one-out stability of T7–T8 and FC5–F3 findings—rather than from the absolute number of nominally significant correlations, which was comparable between ipsilateral and contralateral pairs.

### 4.2 Connectivity–EMG associations in the absence of group-level EEG changes

Although no significant group-level EEG connectivity changes were observed, several connectivity change scores were associated with EMG changes at the individual level. This pattern likely reflects substantial inter-individual variability in stimulation responsiveness, a phenomenon commonly reported in neuromodulation research (Ridding and Ziemann, 2010; López-Alonso et al., 2014).

### 4.3 Interpretation of EMG spectral changes

A noteworthy finding was the significant reduction in right multifidus mean frequency (MF) and high-frequency spectral power (High%) following stimulation (both *p* = 0.004, r_rb = 1.000). Because no sham condition was included, it remains unclear whether these spectral changes reflect a stimulation-specific effect or non-specific temporal factors.

The direction of EEG–EMG associations varied across outcome measures: some negative (connectivity decrease with EMG spectral increase) and some positive (connectivity increase with RMS increase). The physiological basis of this mixed pattern is uncertain.

The physiological basis of these associations remains uncertain. Stimulation may have altered the organisation of distributed cortical networks involved in postural motor control, with reduced inter-regional coupling reflecting network reconfiguration rather than diminished neural activity. Alternatively, gamma–theta stimulation may have shifted inhibitory–excitatory balance within cortical circuits, thereby modulating corticospinal output and motor unit recruitment (Ziemann et al., 1996; Di Lazzaro et al., 1998; Iaccarino et al., 2016).

No measures of corticospinal excitability, corticospinal transmission, or behavioural motor performance were obtained, and these interpretations therefore remain speculative pending replication with more targeted experimental designs.

### 4.4 Ipsilateral predominance and trunk motor control

Most significant EEG–EMG associations observed in the present study involved left-sided trunk muscles, particularly the left multifidus. Although several contralateral relationships were also identified, ipsilateral associations were more numerous and generally demonstrated greater robustness in leave-one-out analyses.

The multifidus muscle plays a critical role in segmental spinal stabilization and postural control (MacDonald et al., 2006; Hodges and Danneels, 2019). Unlike many distal limb muscles that are frequently examined in cortico-muscular coherence studies, trunk muscles receive bilateral cortical and subcortical inputs and operate within highly distributed postural control networks (Strutton et al., 2004; Tsao et al., 2008; Chiou et al., 2016). Consequently, strict hemispheric lateralization would not be expected.

The data suggest a pattern of relative hemispheric predominance rather than strict hemispheric exclusivity. One possible explanation is that stimulation-induced changes within left frontal–temporal networks may have exerted a stronger influence on the regulation of trunk motor output in this sample. In right-handed individuals, the left hemisphere is typically dominant for motor planning and motor control (Knecht et al., 2000), which may contribute to the stronger network-level associations observed in the left hemisphere.

Because all participants were right-handed and the sample size was limited, the apparent asymmetry requires confirmation in larger cohorts. Future studies involving larger cohorts, left-handed participants, and task-specific connectivity analyses will be necessary to determine whether the observed lateralization reflects a reproducible neurophysiological phenomenon or a sample-specific characteristic (Schade et al., 2012; Konieczny et al., 2022).

### 4.5 Robustness of mid% and RMS associations

An important observation emerging from the leave-one-out analyses was that associations involving Mid% spectral power and RMS amplitude were considerably more robust than those involving MF or High% power.

RMS amplitude represents an integrated measure of overall muscle activation and is generally less sensitive to small fluctuations in spectral composition. Similarly, Mid% power reflects activity distributed across a relatively broad frequency range and may therefore provide a more stable representation of motor unit recruitment patterns (Merletti and Parker, 2005; Farina et al., 2010).

In contrast, MF and High% are derived from more specific aspects of the EMG power spectrum and may be more susceptible to inter-individual variability, electrode placement differences, and small changes in signal quality (Merletti and Farina, 2016). This interpretation is consistent with the observation that correlations involving MF and High% were more strongly influenced by the exclusion of individual participants.

Accordingly, stimulation-related EEG–EMG relationships may be more reliably detected using broader measures of muscle activation and spectral distribution than using highly specific spectral indices. This interpretation requires confirmation in larger datasets.

### 4.6 Clinical implications for MCI

These findings may be relevant for understanding motor function in individuals with MCI. Motor impairments, including postural instability and gait disturbances, are increasingly recognised in this population (Montero-Odasso et al., 2012, 2014; Beauchet et al., 2016). The observation that gamma–theta stimulation may influence cortical networks associated with trunk muscle activity suggests that neuromodulation approaches may contribute to interventions targeting both motor and cognitive function (Iaccarino et al., 2016; Spooner et al., 2022).

The predominantly lateralized pattern of EEG–EMG associations observed in this study may inform future research on individualised neuromodulation strategies. If certain cortical networks respond more consistently to stimulation, targeting these networks may enhance intervention efficacy. A randomised controlled trial comparing gamma–theta stimulation with sham control, incorporating both cognitive and motor outcome measures, would represent a logical next step toward translating these preliminary findings into clinical practice.

Finally, the finding that functional connectivity changes, rather than single-channel power changes, were associated with muscle adaptations suggests that network-level EEG measures may serve as sensitive indicators of neurophysiological changes following stimulation (Fox et al., 2014; Bassett and Sporns, 2017). Such metrics may prove useful for monitoring treatment responses and stratifying patients most likely to benefit from neuromodulation in future studies.

### 4.7 Limitations and future directions

Several limitations should be considered when interpreting the present findings.

First, the study included a small sample of nine participants and should therefore be regarded as exploratory. The limited sample size reduced statistical power and increased susceptibility to individual variability (Button et al., 2013). Although leave-one-out analyses were conducted to evaluate robustness, replication in larger cohorts remains necessary.

Second, participants were operationally classified as having MCI based on MMSE scores rather than a comprehensive neuropsychological assessment (Petersen et al., 2009; Albert et al., 2011). Consequently, the sample should be considered a clinically defined cohort rather than a research-diagnostic MCI population meeting the full Petersen criteria.

Third, no sham stimulation condition was included. As a result, stimulation-related changes cannot be definitively distinguished from test–retest effects, habituation, fatigue, or spontaneous physiological variability. The observed EEG–EMG associations should therefore be interpreted as evidence of coordinated physiological changes occurring after stimulation rather than proof of a causal stimulation effect.

Fourth, EEG and EMG were not recorded simultaneously (Mima and Hallett, 1999; Ushiyama et al., 2017). EEG and EMG measurements were also obtained following separate stimulation sessions. Although the two sessions were identical and separated by a three-hour interval intended to reduce carryover effects, cumulative or interactive effects cannot be excluded. Simultaneous EEG–EMG recordings will be required to establish temporal relationships between cortical and muscular responses.

Fifth, the primary connectivity measure was based on Spearman correlation and therefore remains susceptible to volume conduction effects (Bastos and Schoffelen, 2016). Although supplementary analyses using PLI and iCoh were performed to address this concern, these analyses were exploratory and should not be considered definitive validation of the primary findings.

Sixth, all participants were right-handed older adults. The extent to which the observed lateralization patterns generalize to left-handed individuals, younger populations, or patients with more advanced cognitive impairment remains unknown (Knecht et al., 2000; Schade et al., 2012).

Finally, multiple-comparison correction was not applied because of the exploratory nature of this pilot study. The full correlation matrix comprised 6 EEG pairs × 16 EMG variables (96 comparisons), and the number of nominally significant associations (12) exceeded the approximately five associations expected by chance alone at *α* = 0.05. Consequently, some reported associations may represent false-positive findings and should be interpreted cautiously until replicated in independent samples with pre-registered hypotheses.

Future work should incorporate larger samples, sham-controlled designs, simultaneous EEG–EMG acquisition, and frequency-specific connectivity measures to clarify the neurophysiological mechanisms underlying stimulation-related brain–muscle interactions.

## 5 Conclusion

This exploratory pilot study investigated relationships between stimulation-induced changes in resting-state EEG connectivity and trunk muscle activity in older adults operationally classified as having mild cognitive impairment (Albert et al., 2011; Jack et al., 2018).

Although no predefined EEG connectivity pair demonstrated a significant group-level change following stimulation, several connectivity change measures were associated with concurrent changes in multifidus and lumbar paraspinal EMG variables. The most robust associations involved left multifidus Mid% spectral power and RMS amplitude, both of which remained stable across leave-one-out sensitivity analyses.

The observed pattern was characterized by a predominance of ipsilateral EEG–EMG relationships, accompanied by a smaller number of contralateral associations, suggesting relative hemispheric predominance rather than strict hemispheric exclusivity (Knecht et al., 2000; Strutton et al., 2004).

Supplementary phase-based analyses provided limited support for the possibility that some EEG–EMG relationships may not be entirely explained by volume conduction effects, although these findings require confirmation in larger studies.

These results provide initial evidence that gamma–theta stimulation may influence coordinated brain–muscle responses in older adults with cognitive impairment (Iaccarino et al., 2016; Spooner et al., 2022). However, the exploratory nature of the study, the small sample size, and the absence of a sham control condition require that all findings be considered hypothesis-generating (Button et al., 2013). Confirmation in larger, controlled studies will be necessary to determine the physiological significance and clinical relevance of these associations.

## Data Availability

The datasets presented in this study can be found in online repositories. The data are available at: Yao, Runhong (2026), ‘Hemisphere-specific EEG–EMG associations following gamma–theta stimulation in mild cognitive impairment,’ Mendeley Data, V1, doi: https://doi.org/10.17632/8zp3gdmvmk.1.
