# Supplementary material for: Predominantly left-lateralized EEG–EMG associations following gamma–theta stimulation in older adults with mild cognitive impairment
Source: Front Aging Neurosci. 2026 Jul 17;18:1882681. doi: 10.3389/fnagi.2026.1882681 (PMC13423906; doi:10.3389/fnagi.2026.1882681)
Supplement: Supplementary file 1 [file Data_Sheet_1.DOCX]

Supplementary Material

All supplementary analyses are exploratory and intended to provide transparency regarding the stability and specificity of the observed EEG–EMG associations. No correction for multiple comparisons was applied.

# Supplementary Table

**Supplementary Table S1. Complete significant EEG–EMG associations from the exploratory full correlation matrix (6 EEG pairs × 16 EMG variables, N = 9)**

| **EEG pair (Δr)** | **EMG variable (Δ)** | **Association type** | **ρ** | **p** |  |
| --- | --- | --- | --- | --- | --- |
| **Ipsilateral and bilateral associations** | | | | | |
| FC5–F3 | L. Mult. RMS | Ipsilateral / left | **+0.800** | **0.010** | ****** |
| T7–T8 | L. Mult. Mid% | Ipsilateral / left | **−0.800** | **0.010** | ****** |
| FC5–F3 | L. Mult. MF | Ipsilateral / left | −0.683 | 0.042 | ***** |
| T7–T8 | L. Mult. MF | Ipsilateral / left | −0.683 | 0.042 | ***** |
| FC5–F3 | L. Mult. High% | Ipsilateral / left | −0.667 | 0.050 | ***** |
| F4–T8 | R. LP Mid% | Ipsilateral / right | −0.667 | 0.050 | ***** |
| **Contralateral associations** | | | | | |
| FC6–F4 | L. LP MF | Contralateral (R→L) | **+0.800** | **0.010** | ****** |
| F4–T8 | L. Mult. Mid% | Contralateral (R→L) | −0.783 | 0.013 | ***** |
| T7–T8 | R. LP Mid% | Contralateral (L→R) | −0.750 | 0.020 | ***** |
| T7–T8 | R. Mult. Mid% | Contralateral (L→R) | +0.733 | 0.025 | ***** |
| FC5–F3 | R. Mult. RMS | Contralateral (L→R) | +0.700 | 0.036 | ***** |
| FC6–F4 | L. LP High% | Contralateral (R→L) | +0.700 | 0.036 | ***** |

*Note.* Note. EEG variables represent connectivity change scores (Δr = rpost − rpre) for the six predefined electrode pairs.EMG variables represent change scores (Δ = post − pre). Associations are grouped according to the predominant hemispheric relationship between EEG connectivity and EMG activity. All p-values are unadjusted. Because of the exploratory nature of the study and the small sample size (N = 9), results should be considered hypothesis-generating.

**Supplementary Table S2. Single-channel EEG amplitude change vs. EMG change: significant and trend-level associations**

| **EEG Channel**  **(Δ mean voltage, μV)** | **EMG Variable (Δ)** | **ρ** | **p** |
| --- | --- | --- | --- |
| **Nominally significant associations (p ≤ 0.05)** | | | |
| **FC5** | L. Mult. RMS | **-0.683** | **0.042** |
| **AF4** | R. Mult. RMS | **-0.683** | **0.042** |
| **F8** | R. Mult. RMS | **-0.667** | **0.050** |
| **Trend-level associations (0.05 ≤ p < 0.20, \|ρ\| ≥ 0.55)** | | | |
| AF3 | L. Mult. RMS | -0.650 | 0.058 |
| T8 | L. Mult. RMS | -0.633 | 0.067 |
| P7 | L. Mult. RMS | -0.617 | 0.077 |
| FC5 | L. LP RMS | -0.583 | 0.099 |
| O1 | L. Mult. RMS | -0.583 | 0.099 |
| T7 | L. Mult. RMS | -0.583 | 0.099 |
| F4 | R. Mult. RMS | -0.567 | 0.112 |
| AF3 | L. LP High% | +0.550 | 0.125 |
| F3 | R. Mult. MF | -0.550 | 0.125 |

*Note.* Single-channel EEG measure = change in mean raw voltage (Δ = post − pre, μV) of the 0.5–45 Hz band-pass filtered signal, used as a simple index of overall signal level. EMG variables: RMS = root mean square amplitude; MF = mean frequency (Hz); High% = 60–100 Hz spectral power proportion. Low-frequency spectral power (< 20 Hz) was excluded from EMG analyses because signals were band-pass filtered between 20 and 100 Hz. Nominally significant associations were primarily observed for RMS-related EMG variables and involved multiple frontal and temporal electrode sites; however, these findings should be interpreted cautiously given the exploratory nature of the analysis and the absence of multiple comparison correction. p-values are unadjusted. Trend-level entries are limited to |ρ| ≥ 0.55. The complete single-channel correlation matrix is provided in Figure S2.

**Supplementary Table S3. Individual participant EEG connectivity change scores (Δr = r_post − r_pre) for the six pre-specified electrode pairs (N = 9)**

| **Subject** | **F4–T8 Δr** | **FC6–F4 Δr** | **AF3–F3 Δr** | **FC5–F3 Δr** | **O1–O2 Δr** | **T7–T8 Δr** |
| --- | --- | --- | --- | --- | --- | --- |
| **S1** | -0.299 | **-0.332** | -0.237 | -0.194 | **-0.523** | **-0.338** |
| **S2** | **+0.337** | +0.228 | +0.001 | +0.068 | -0.140 | +0.016 |
| **S3** | +0.037 | -0.028 | -0.101 | +0.138 | +0.040 | **+0.359** |
| **S4** | +0.244 | +0.184 | +0.006 | +0.060 | -0.137 | +0.090 |
| **S5** | **-0.370** | **+0.591** | -0.228 | **-0.507** | **+0.395** | -0.059 |
| **S6** | +0.270 | -0.119 | -0.013 | -0.169 | **+0.315** | +0.288 |
| **S7** | +0.012 | +0.123 | **+0.323** | +0.138 | +0.144 | -0.047 |
| **S8** | +0.019 | **+0.417** | -0.182 | +0.049 | **-0.528** | +0.039 |
| **S9** | -0.032 | +0.005 | -0.006 | +0.066 | +0.004 | **-0.386** |
| **Mean** | **+0.024** | **+0.119** | **-0.049** | **-0.039** | **-0.048** | **-0.004** |
| **SD** | **0.242** | **0.279** | **0.171** | **0.213** | **0.326** | **0.248** |

*Note.* Δr = post-intervention Spearman correlation coefficient minus pre-intervention Spearman correlation coefficient for each electrode pair. Positive values indicate increased inter-electrode signal coupling following stimulation; negative values indicate decreased coupling. Teal shading denotes Δr > +0.30 (increased coupling); coral shading denotes Δr < −0.30 (decreased coupling). Mean and SD are provided for descriptive purposes. No statistically significant group-level changes were observed (all Wilcoxon signed-rank tests: p > 0.25; see Table 2 in main text).

**Supplementary Table S4. Nominally significant exploratory EEG–EMG associations identified using phase-lag index (PLI) and imaginary coherence (iCoh) (uncorrected p < 0.05, N = 9)**

| **EEG metric (Δ)** | **EMG variable (Δ)** | **Band** | **ρ** | **p** |  |
| --- | --- | --- | --- | --- | --- |
| **Associations involving electrode pairs also highlighted in the primary Spearman analysis** | | | | | |
| **FC6–F4 gamma iCoh** | R. LP MF | γ | **-0.917** | **0.001** | ****** |
| **FC5–F3 gamma PLI** | R. LP High% | γ | **+0.845** | **0.004** | ****** |
| **F4–T8 gamma PLI** | L. Mult. Mid% | γ | **-0.828** | **0.006** | ****** |
| **T7–T8 gamma PLI** | L. LP Mid% | γ | -0.733 | 0.025 | ***** |
| **T7–T8 theta iCoh** | L. LP RMS | θ | +0.700 | 0.036 | ***** |
| **FC6–F4 gamma iCoh** | R. Mult. Mid% | γ | +0.717 | 0.030 | ***** |
| **F4–T8 theta PLI** | R. LP MF | θ | -0.683 | 0.042 | ***** |
| **Additional associations involving other electrode pairs** | | | | | |
| O1–O2 theta iCoh | R. LP RMS | θ | **-0.800** | **0.010** | ****** |
| O1–O2 gamma PLI | L. Mult. RMS | γ | -0.783 | 0.013 | ***** |
| O1–O2 theta PLI | L. Mult. Mid% | θ | -0.766 | 0.016 | ***** |
| AF3–F3 theta PLI | R. Mult. RMS | θ | -0.714 | 0.031 | ***** |
| AF3–F3 theta PLI | L. Mult. Mid% | θ | +0.689 | 0.040 | ***** |

*Note.* PLI = phase-lag index; iCoh = imaginary part of coherence. Gamma band: 27–45 Hz; theta band: 4–8 Hz. These analyses were conducted as exploratory sensitivity analyses to evaluate whether EEG–EMG associations could also be detected using connectivity metrics less susceptible to volume conduction artifacts. Results are not directly comparable to the primary Spearman-based connectivity analysis, which was based on broadband temporal signal coupling rather than narrow-band phase relationships. Because of the small sample size (N = 9) and multiple comparisons, all results should be considered nominally significant and hypothesis-generating only. No correction for multiple comparisons was applied. ** p < 0.01; * p < 0.05. Associations involving electrode pairs that also appeared in the primary EEG–EMG analyses are listed in the upper section of the main text. γ = gamma (27–45 Hz); θ = theta (4–8 Hz). EMG variables: RMS = root mean square amplitude; MF = mean frequency (Hz); Mid% = 20–60 Hz power proportion; High% = 60–100 Hz power proportion.

**Supplementary Table S5. Sensitivity analysis using Fisher z-transformed EEG connectivity change scores (Δz = z_post − z_pre, N = 9)**

| **EEG pair (Δz)** | **EMG variable (Δ)** | **ρ (Δz)** | **p (Δz)** | **ρ (Δr)** | **p (Δr)** |  | **Dir.** |
| --- | --- | --- | --- | --- | --- | --- | --- |
| **Associations significant with both Δz and Δr (7 of 12 originally significant EEG–EMG associations)** | | | | | | |  |
| FC6–F4 | L. LP MF | **+0.850** | **0.004** | +0.800 | 0.010 | ****** | **✓** |
| FC5–F3 | L. Mult. RMS | **+0.817** | **0.007** | +0.800 | 0.010 | ****** | **✓** |
| T7–T8 | L. Mult. Mid% | **-0.767** | **0.016** | -0.800 | 0.010 | ****** | **✓** |
| F4–T8 | L. Mult. Mid% | -0.767 | 0.016 | -0.783 | 0.013 | ***** | **✓** |
| T7–T8 | R. Mult. Mid% | +0.717 | 0.030 | +0.733 | 0.025 | ***** | **✓** |
| FC6–F4 | L. LP High% | +0.750 | 0.020 | +0.700 | 0.036 | ***** | **✓** |
| FC5–F3 | L. Mult. High% | -0.783 | 0.013 | -0.667 | 0.050 | ***** | **✓** |
| **Associations significant with Δr only (p < 0.05 for Δr; p ≥ 0.05 for Δz)** | | | | | | |  |
| T7–T8 | R. LP Mid% | −0.650 | 0.059 | -0.750 | 0.020 | ***** | **✓** |
| FC5–F3 | R. Mult. RMS | +0.583 | 0.099 | +0.700 | 0.036 | ***** | **✓** |
| FC5–F3 | L. Mult. MF | -0.633 | 0.068 | -0.683 | 0.042 | ***** | **✓** |
| T7–T8 | L. Mult. MF | -0.617 | 0.077 | -0.683 | 0.042 | ***** | **✓** |
| F4–T8 | R. LP Mid% | -0.617 | 0.077 | -0.667 | 0.050 | ***** | **✓** |
| **Association significant with Δz only** | | | | | | |  |
| FC5–F3 | R. Mult. High% | -0.733 | 0.025 | -0.617 | 0.077 | ***** | **✓** |

*Note.* Fisher z-transformation was applied to all Spearman correlation coefficients prior to computing change scores: Δz = z_post − z_pre, where z = 0.5 × ln[(1+r)/(1−r)]. Correlation analyses using Δz were then performed using Spearman rank correlation against all EMG change variables. The Δr (primary) and Δz (sensitivity) results are shown side by side for comparison. 7 of the 12 associations significant with Δr were also significant with Δz, and the direction of association was consistent in all cases, indicating that the primary findings were not an artifact of the difference-score metric. 5 associations reaching significance with Δr did not reach nominal significance with Δz (p range 0.059–0.099), although effect directions remained unchanged in all cases. ** p < 0.01; * p < 0.05. Δr values are from Table S1; p-values unadjusted throughout.

# Supplementary Figures


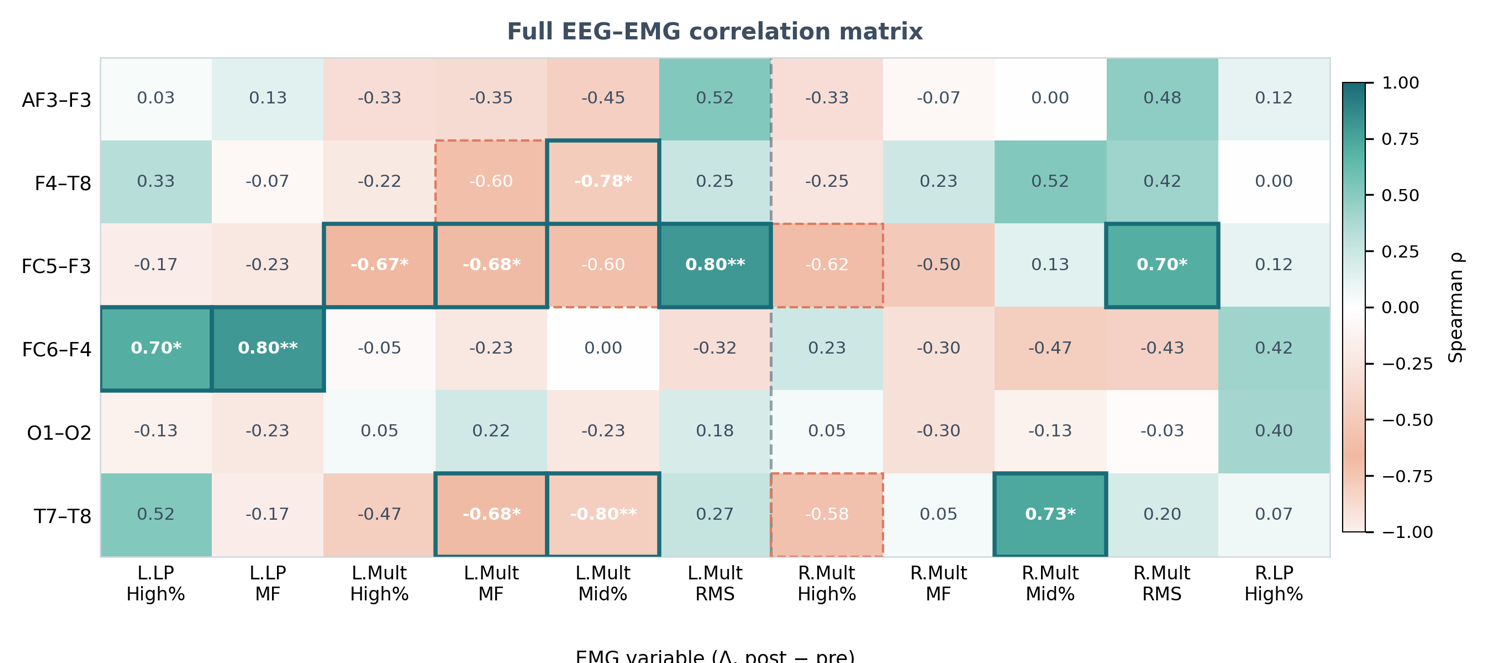


**Supplementary Figure S1.** Full EEG–EMG Spearman correlation matrix. Heatmap of Spearman rank correlation coefficients (ρ) for all combinations of six EEG connectivity change scores (rows) and EMG change variables (columns; N = 9). EEG rows represent connectivity change scores (Δr) for the six predefined electrode pairs. EMG columns include RMS amplitude, mean frequency (MF), mid-frequency power (Mid%, 20–60 Hz), and high-frequency power (High%, 60–100 Hz) for left and right multifidus and lumbar paraspinal muscles. Low-frequency spectral power (< 20 Hz) was excluded because EMG signals were band-pass filtered between 20 and 100 Hz. Cell values show Spearman ρ; teal solid borders indicate p < 0.05 and dashed orange borders indicate p < 0.10. Cell shading reflects correlation magnitude according to the colour scale (teal = positive, coral = negative). A complete list of all nominally significant associations is provided in Supplementary Table S1. * p < 0.05; ** p < 0.01; † p < 0.10. p-values are unadjusted.


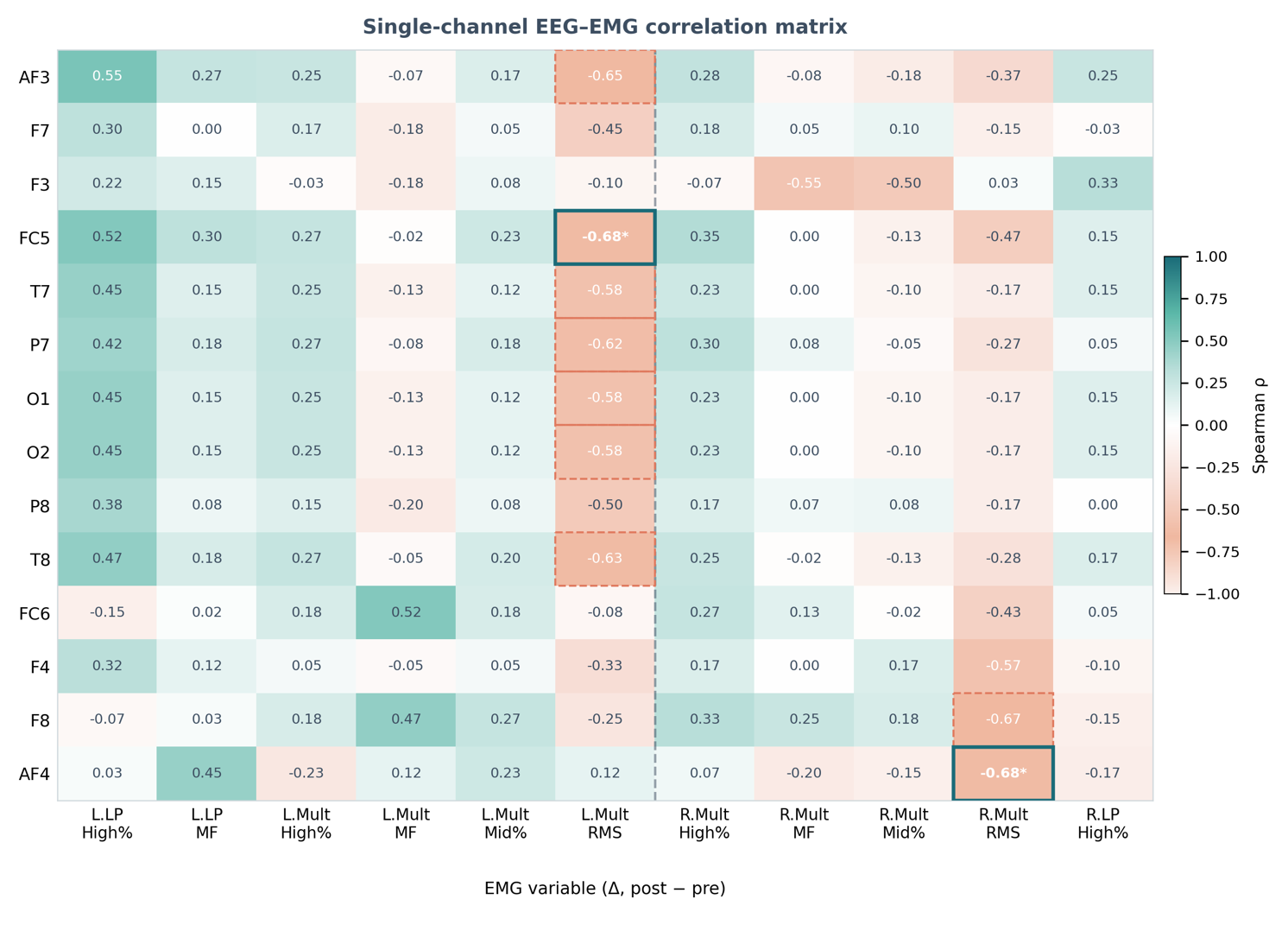


**Supplementary Figure S2.** Single-channel EEG–EMG correlation matrix. Heatmap of Spearman rank correlation coefficients (ρ) between single-channel EEG amplitude change scores (rows; Δ mean voltage, μV; 14 channels) and EMG change variables (columns; N = 9). Single-channel EEG measures represent the change in mean raw voltage of the band-pass filtered signal (0.5–45 Hz) at each electrode site, used as a simple index of overall signal level. EMG variables are identical to those in Figure S1. Cell values show Spearman ρ; teal borders indicate p ≤ 0.05; dashed orange borders indicate p < 0.10. Only three associations reached nominal significance (p ≤ 0.05), all involving RMS-related EMG variables. Cell shading reflects correlation magnitude according to the colour scale. p-values are unadjusted. Selected nominally significant and trend-level associations are listed in Supplementary Table S2.


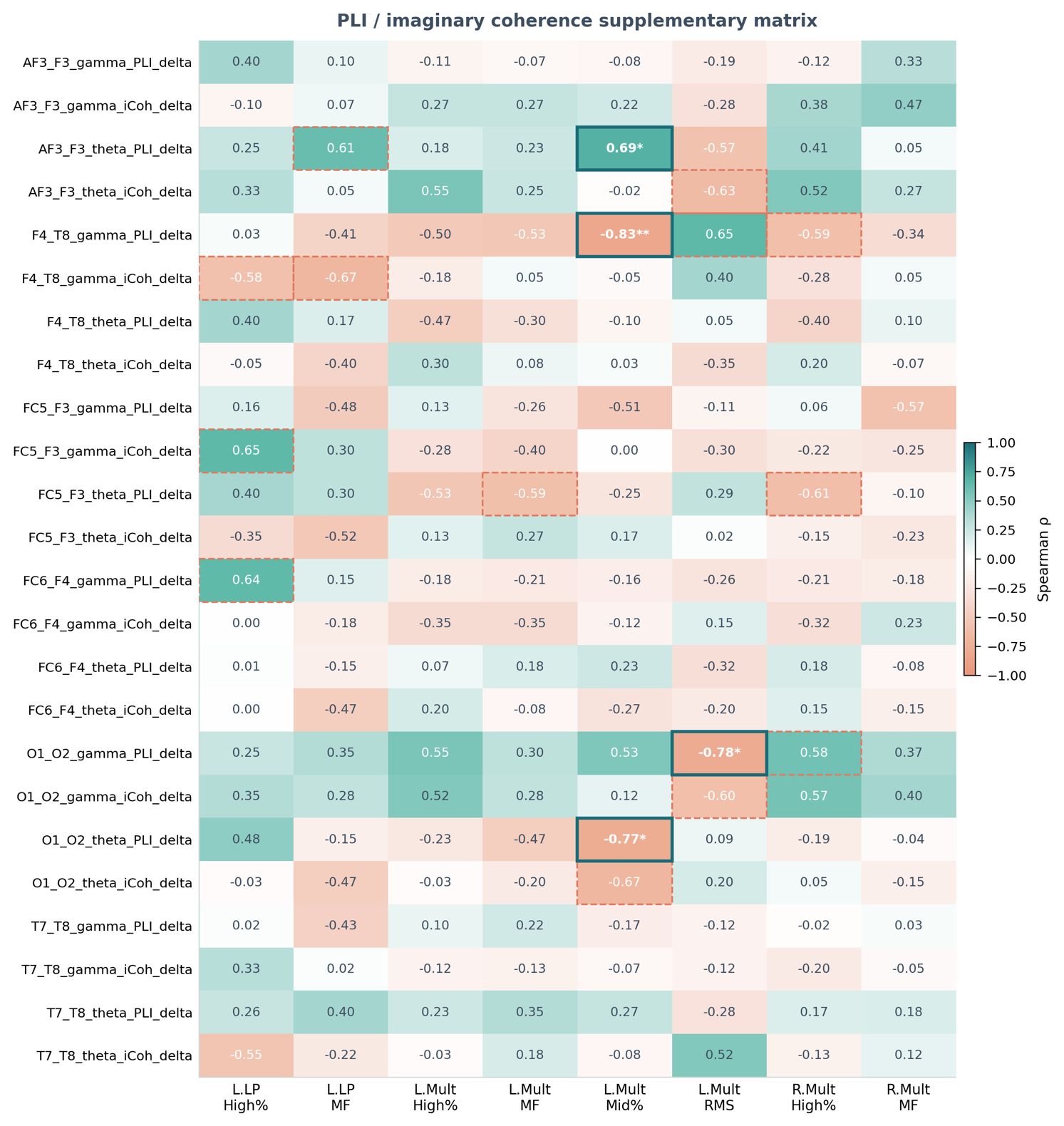


**Supplementary Figure S3.** PLI / imaginary coherence supplementary matrix. Heatmap of Spearman rank correlation coefficients (ρ) between phase-based EEG connectivity change scores (rows) and EMG change variables (columns; N = 9). EEG rows represent change scores (Δ = post − pre) for phase-lag index (PLI) and imaginary coherence (iCoh) computed within theta (4–8 Hz) and gamma (27–45 Hz) frequency bands for each of the six predefined electrode pairs. PLI and iCoh are less sensitive to zero-lag volume conduction artifacts than broadband Spearman correlation and were computed as supplementary sensitivity analyses. Cell values show Spearman ρ. Teal borders indicate p < 0.05; dashed orange borders indicate p < 0.10. Cell shading reflects correlation magnitude according to the colour scale (teal = positive, coral = negative). p-values are unadjusted. These analyses are exploratory; see Supplementary Table S4 for a complete list of nominally significant associations and Methods 2.3 for analytical details. * p < 0.05; ** p < 0.01.
